# Case report: Salvage capmatinib therapy in *KIF5B-MET* fusion-positive lung adenocarcinoma with resistance to telisotuzumab vedotin

**DOI:** 10.3389/fonc.2022.919123

**Published:** 2022-08-11

**Authors:** Chien-Yu Lin, Sheng-Huan Wei, Yi-Lin Chen, Chung-Ta Lee, Shang-Yin Wu, Chung-Liang Ho, Dean C. Pavlick, Po-Lan Su, Chien-Chung Lin

**Affiliations:** ^1^ Department of Internal Medicine, National Cheng Kung University Hospital, College of Medicine, National Cheng Kung University, Tainan, Taiwan; ^2^ Department of Pathology, National Cheng Kung University Hospital, College of Medicine, National Cheng Kung University, Tainan, Taiwan; ^3^ Department of Oncology, National Cheng Kung University Hospital, College of Medicine, National Cheng Kung University, Tainan, Taiwan; ^4^ Department of Research and Development, Foundation Medicine, Inc., Cambridge, MA, United States; ^5^ Institute of Clinical Medicine, National Cheng Kung University Hospital, College of Medicine, National Cheng Kung University, Tainan, Taiwan; ^6^ Department of Biochemistry and Molecular Biology, College of Medicine, National Cheng Kung University, Tainan, Taiwan

**Keywords:** *KIF5B-MET* fusion, lung adenocarcinoma, capmatinib, telisotuzumab vedotin, case report

## Abstract

Telisotuzumab vedotin is a *MET*-targeting antibody–drug conjugate that has demonstrated a good treatment response in patients with *EGFR* wild-type MET-overexpressing non-squamous non-small cell lung cancer. However, patients have been reported to acquire resistance to this drug, and the subsequent therapy has not been standardized. Here, we present a case of a 56-year-old woman diagnosed with *KIF5B-MET* fusion-positive non-small cell lung cancer who had a durable response to capmatinib after acquired resistance to telisotuzumab vedotin.

## Introduction

Telisotuzumab vedotin, previously named ABBV-399, is an antibody-drug conjugate, which comprises a human MET-targeting antibody, ABT-700, and a cytotoxic microtubule inhibitor, monomethyl auristatin E, through a valine-citrulline linker (1). Preliminary results from a phase 2 trial demonstrated that telisotuzumab vedotin yielded an objective response rate of 53.8% in patients with epidermal growth factor receptor (*EGFR*) wild-type MET-overexpressing non-squamous non-small cell lung cancer (NSCLC) (2). However, the subsequent treatment strategy after acquiring resistance to telisotuzumab vedotin remains under investigation. Here, we present the case of a patient with *KIF5B-MET* fusion-positive advanced NSCLC who exhibited a durable response to capmatinib after acquiring resistance to telisotuzumab vedotin. To the best of our knowledge, this is the first case report to describe the clinical benefit of capmatinib in patients with NSCLC with *MET* fusion.

## Case presentation

**Figure 1:**
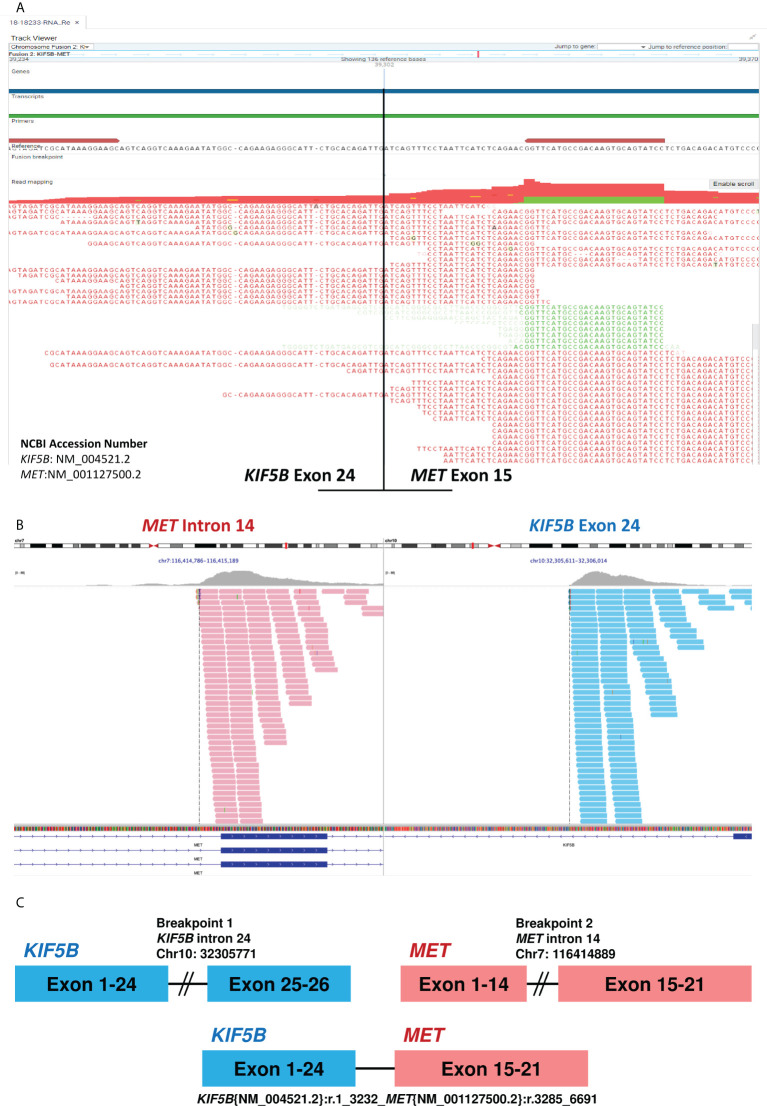
Next-generation sequencing of tissue before and after Telisotuzumab Vedotin. **(A)** The RNA-based NGS by QIAact Lung All-in-One assay revealed a *KIF5B-MET* (K24;M15) fusion. **(B)** The DNA-based NGS by FoundationOne^®^ CDx after Telisotuzumab Vedotin still revealed *KIF5B-MET* fusion. The data was provided by the Department of Research and Development, Foundation Medicine Inc. **(C)** The diagram of *KIF5B-MET* fusion. NGS, next-generation sequencing.

In October 2019, a 56-year-old woman was diagnosed with poorly differentiated stage IIIC pulmonary adenocarcinoma. The tumor involved the right upper lobe and mediastinal lymph nodes. Polymerase chain reaction analysis revealed wild-type *EGFR*, and the immunohistochemical staining for *ALK* and *ROS1* was negative. Concurrent chemoradiotherapy followed by 12-month durvalumab consolidation was administered, and the tumor showed significant shrinkage. Eleven months after completion of durvalumab therapy, the patient experienced disease progression with enlargement of the left axillary lymph node. Sonography-guided biopsy revealed an adenocarcinoma. Next-generation sequencing (NGS) by the QIAact Lung All-in-One assay revealed *KIF5B-MET* fusion (**Figure 1A**), *PDGFRA* mutation, and *RICTOR* amplification (**Table 1**). She was then enrolled in a phase 2 clinical trial with telisotuzumab vedotin, and subsequent chest computed tomography revealed partial regression of the axillary lymph node. During the administration of telisotuzumab vedotin, the patient experienced grade 2 blurred vision and grade 1 pneumonitis.

**Table 1:** Results from NGS testing before and after Telisotuzumab Vedotin.

Before Telisotuzumab Vedotin*		After Telisotuzumab Vedotin**	
**Gene**	**Genomic alteration**	**Gene**	**Genomic alteration**
***MET***	KIF5B-MET fusion (AF: 6.25%)	***MET***	KIF5B-MET fusion
***MET***	Amplification	***PIK3CA***	amplification
***PDGFRA***	D842A (AF: 5.02%)	***CCNE1***	amplification
***RICTOR***	Amplification	***KEL***	rearrangement intron 14
		***MUTYH***	splice site 892-2A>G
		***TP53***	M169fs*16

*Next-generation sequencing testing was performed by QIAact Lung All-in-One assay and revealed wild-type sequences in hotspots of the following genes: ALK, AKT1, DDR2, EGFR, ERBB2, ESR1, FGFR1, KRAS, KIT, MAP2K1, MET, NRAS, NTRK1, PDGFRA, PIK3CA, PTEN, and ROS1.

**Next-generation sequencing testing was performed by FoundationOne^®^ CDx.

**Figure 2:**
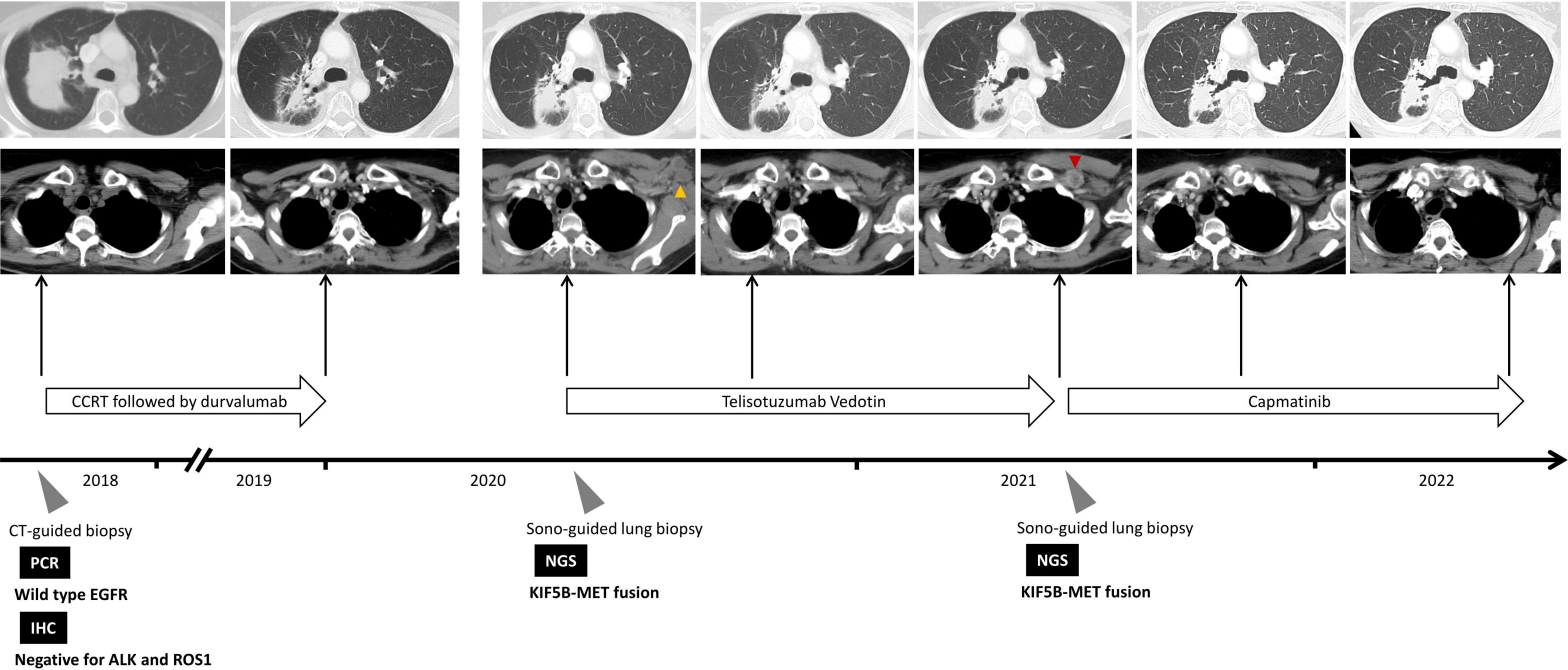
Summary of treatment courses mentioned in this case report. The yellow arrowhead indicate the target lesion at disease progression after consolidation therapy with durvalumab, whereas the red arrowhead indicates the target lesion at disease progression after the use of Telisotuzumab Vedotin. *ALK*, anaplastic lymphoma kinase; CCRT, concurrent chemoradiotherapy; CT, computed tomography; *EGFR*, epidermal growth factor receptor; IHC, immunohistochemical; NGS, next-generation sequencing; PCR, polymerase chain reaction; *ROS1*, ROS proto-Oncogene 1.

However, 8 months later, the patient experienced disease progression again with new-onset left chest wall metastasis. Re-biopsy showed adenocarcinoma, and NGS using FoundationOne^®^ CDx revealed *KIF5B-MET* fusion without other oncogenic driver mutations (**Figure 1B** and **Table 1**), which implies a potential response to *MET* tyrosine kinase inhibitor (TKI). Thus, capmatinib was administered, and further imaging showed a dramatic response after 3 months of therapy. The therapeutic response was determined based on the radiographic evidence according to Response Evaluation Criteria in Solid Tumors version 1.1 (3). The patient has been on capmatinib for more than nine months, and no significant adverse events have developed. The treatment course is summarized in **Figure 2**.

## Discussion

*MET* fusion is a rare oncogenic driver mutation in NSCLC, which comprises only 0.5% of the total number of patients with lung cancer (4, 5). This may be underestimated if the genomic test is carried out using DNA-based NGS (6). Several fusion of *MET* gene partners have been described in lung adenocarcinomas, including *KIF5B*, *HLA-DRB1*, *UBE2H*, *CD47*, *ATXN7L1*, *SPECC1L*, and *CAV1* (7, 8). Similar to the *MET* exon 14 skipping mutation, *MET* fusion induces ligand-independent activation of downstream signaling pathways, resulting in cellular proliferation, survival, migration, and angiogenesis (6). In addition, depending on the breakpoint of each fusion gene, *MET* fusion might also result in overexpression of MET protein on the cell surface. In a study conducted by Gow et al., there were two NSCLC cases with the *KIF5B-MET* rearrangement, which is a fusion between exons 1-24 of *KIF5B* and exons 15-21 of *MET*. The loss of exon 14 in *MET* results in a lack of the juxtamembrane domain of the MET protein, leading to its overexpression during immunohistochemical staining, which is secondary to failure of ubiquitin-dependent protein degradation (4). Our patient also harbored a *KIF5B-MET* rearrangement with a fusion between exons 1-24 of *KIF5B* and exons 15-21 of *MET* (**Figure 1C**), which is associated with MET protein overexpression and a potentially higher response rate to telisotuzumab vedotin (2). In addition, the *KIF5B-MET* rearrangement also contain the exon 1-15 of the *KIF5B*, which preserves the kinesin motor and coiled-coil domains that mediate homodimerization and subsequent activation of *MET* signaling pathway (9). The *KIF5B* is also an active promoter, which had been reported to activate and enhance the downstream oncogenic pathway of ALK and RET (10, 11).

The therapeutic strategy after acquiring resistance to telisotuzumab vedotin remains to be standardized. In our case report, NGS of the re-biopsied tumor revealed *KIF5B-MET* fusion, and no other oncogenic driver mutation was discovered, which implies that the tumor might be responsive to targeted therapy. In a *in vivo* study, the lung cancer xenograft model of *KIF5B-MET* fusion exhibited a good treatment response to crizotinib, a type Ia *MET*-TKI (4). Several case reports also demonstrated a good treatment response to crizotinib among NSCLC patients with *de noo MET* fusion (5, 8, 12–15). In addition, *MET* fusion could also be a resistance mechanism in patients with *EGFR*-mutant NSCLC who experience disease progression after treatment with *EGFR*-TKI. The combination of *EGFR*-TKIs and *MET*-TKIs can provide clinical benefits (5, 16, 17). Recently, targeted therapies with type Ib *MET*-TKIs, including tepotinib and capmatinib, have demonstrated their effectiveness and have been approved to treat patients with *MET* exon 14 skipping mutation-positive NSCLC (18). Capmatinib has a higher potency for MET protein binding based on an *in vitro* study (19); however, there are no reports on capmatinib treatment in patients with *MET* fusion. In the present case report, our patient experienced significant tumor shrinkage after receiving capmatinib, which implies that capmatinib could be a potential salvage therapy for patients with NSCLC with *MET* fusion and acquired resistance to telisotuzumab vedotin.

In summary, our case report highlights that patients with *MET* fusion could potentially respond to capmatinib treatment. More importantly, it could be used as salvage therapy for patients with acquired resistance to telisotuzumab vedotin. Further prospective clinical trials are warranted to validate these results.

## Data availability statement

The raw data supporting the conclusions of this article will be made available by the authors, without undue reservation.

## Ethics statement

This study was reviewed and approved by The Review Board and Ethics Committee of National Cheng Kung University Hospital. The patient provided their written informed consent to participate in this study.

## Author contributions

C-YL, S-HW, and P-LS had full access to data in this case report and takes responsibility for the integrity and accuracy of data analysis. Y-LC and C-LH contributed to the genomic data analysis. C-TL, S-YW, C-LH, DP, P-LS, and C-CL contributed to the scientific review and final approval of this manuscript. All authors contributed to the article and approved the submitted version.

## Funding

The present study was funded by grant no. MOST 110-2314-B-006-102, MOST 111-2314-B-006 -092-MY3 from the Ministry of Science and Technology, Taiwan, and grant no. NCKUH-11102019 from National Cheng Kung University Hospital, Taiwan.

## Acknowledgments

The patient involved in this case report gave his informed consent authorizing use and disclosure of his health information. We also thank the Molecular Medicine Core Laboratory, Clinical Medicine Research Center, National Cheng Kung University Hospital for technical support, experimental design, and data analysis with the GeneReader NGS system.

## Conflict of interest

DP is employed by Foundation Medicine Inc.

The remaining authors declare that the research was conducted in the absence of any commercial or financial relationships that could be construed as a potential conflict of interest.

## Publisher’s note

All claims expressed in this article are solely those of the authors and do not necessarily represent those of their affiliated organizations, or those of the publisher, the editors and the reviewers. Any product that may be evaluated in this article, or claim that may be made by its manufacturer, is not guaranteed or endorsed by the publisher.
